# The Big Drink Debate: perceptions of the impact of price on alcohol consumption from a large scale cross-sectional convenience survey in north west England

**DOI:** 10.1186/1471-2458-11-664

**Published:** 2011-08-23

**Authors:** Penny A Cook, Penelope A Phillips-Howard, Michela Morleo, Corinne Harkins, Linford Briant, Mark A Bellis

**Affiliations:** 1 Centre for Public Health, Liverpool John Moores University, Henry Cotton Campus, Liverpool, L3 2ET, UK; 2 Edge Hill University, St Helens Road, Ormskirk, Lancashire, L39 4QP, UK; 3 Department of Engineering Mathematics, University Of Bristol, Queen's Building, University Walk, Bristol, BS8 1TR, UK

## Abstract

**Background:**

A large-scale survey was conducted in 2008 in north west England, a region with high levels of alcohol-related harm, during a regional 'Big Drink Debate' campaign. The aim of this paper is to explore perceptions of how alcohol consumption would change if alcohol prices were to increase or decrease.

**Methods:**

A convenience survey of residents (≥ 18 years) of north west England measured demographics, income, alcohol consumption in previous week, and opinions on drinking behaviour under two pricing conditions: low prices and discounts and increased alcohol prices (either 'decrease', 'no change' or 'increase'). Multinomial logistic regression used three outcomes: 'completely elastic' (consider that lower prices increase drinking and higher prices decrease drinking); 'lower price elastic' (lower prices increase drinking, higher prices have no effect); and 'price inelastic' (no change for either).

**Results:**

Of 22,780 drinkers surveyed, 80.3% considered lower alcohol prices and discounts would increase alcohol consumption, while 22.1% thought raising prices would decrease consumption, making lower price elasticity only (i.e. lower prices increase drinking, higher prices have no effect) the most common outcome (62%). Compared to a high income/high drinking category, the lightest drinkers with a low income (adjusted odds ratio AOR = 1.78, 95% confidence intervals CI 1.38-2.30) or medium income (AOR = 1.88, CI 1.47-2.41) were most likely to be lower price elastic. Females were more likely than males to be lower price elastic (65% vs 57%) while the reverse was true for complete elasticity (20% vs 26%, P < 0.001).

**Conclusions:**

Lower pricing increases alcohol consumption, and the alcohol industry's continued focus on discounting sales encourages higher drinking levels. International evidence suggests increasing the price of alcohol reduces consumption, and one in five of the surveyed population agreed; more work is required to increase this agreement to achieve public support for policy change. Such policy should also recognise that alcohol is an addictive drug, and the population may be prepared to pay more to drink the amount they now feel they need.

## Background

Alcohol accounts for 4% of deaths worldwide [1]. International research demonstrates that increasing the price of alcohol is one of the strongest interventions for reducing consumption and potential harm [2-5]. A meta-analysis of 112 studies demonstrated that for each beverage type, an increase in price was associated with a decrease in consumption [6]. Increasing the price of alcohol leads to reductions in alcohol harm (e.g. liver cirrhosis in the USA;[7] alcohol-related mortality and morbidity in Australia;[8] alcohol poisoning and public order offences in Ireland [9,10]). Conversely, tax cuts, which reduced the price of alcohol in Finland, were associated with increased alcohol-related mortality [11]. However, despite this, the price of alcohol continues to decline in real terms in many European countries [12,13].

Recent surveys in north west (NW) England have shown alcohol to cost as little as £0.14 (€0.16; $0.23) per unit (1 unit = 10 ml or 8 g pure alcohol) in liquor/convenience stores and supermarkets,[14,15] while heavy drinkers in Scotland were able to purchase alcohol as cheaply as £0.09 (€0.10; $0.15) per unit [16]. Modelled data suggest that over 49,000 hospital admissions in England would be prevented per annum after ten years if a minimum price of £0.50 (€0.56; $0.83) per unit were applied [3]. England's then Chief Medical Officer [17] and the National Institute for Health and Clinical Excellence [5] have recommended a minimum price. A minimum price policy has been considered in Scotland [16]. In 2011 The UK government implemented a tax increase for the highest strength beers and a ban on below cost selling,[18] but this is argued to be too trivial for any public health impact [19]. Public engagement in population-level health interventions is vital. However, there is little published information on whether the UK public would support interventions on alcohol price.

Locally-driven interventions provide some preliminary evidence that price modification of alcohol and practical restrictions are a publicly acceptable tool to diminish harmful drinking and would benefit the community [20]. Nevertheless, public perceptions of the problem and the evidence around effective interventions are influenced by the alcohol industry, as is the willingness of governments to increase regulation [19,21]. Lobbying against population-level interventions by the alcohol industry, although strenuous,[22] is not universal [23]. Representatives of the on-licence trade recognise that minimum pricing could benefit their business,[24] and members of the off-licensed trade have vocalised their support [25].

In 2008, the 'Big Drink Debate' (BDD) was conducted in NW England, a region that suffers disproportionately high levels of alcohol-related harm compared with England overall [26-28]. As a large-scale regional survey of the general public, it aimed to consult on alcohol consumption and perceptions of factors relating to alcohol use, generating data for policy reform. This paper focuses on the perceptions of how people would modify their drinking behaviour if the price of alcohol were to either increase or decrease.

## Methods

### Study population

NW England, a region with above average deprivation,[29] has a population of 6.9 million [30]. General rates of perceived good health are lower than average [31] and residents experience the shortest life expectancy in England [32]. The region's alcohol-attributable hospital admissions rate was 2080.7 per 100,000 compared to 1582.7 nationally in 2008/09 [26]. Health harms vary considerably even within the region [26,33]. The BDD targeted adults (≥18 years) normally resident in the region, but excluded non-English speakers as translation into minority languages was impractical for such a large-scale survey.

The BDD was a broad awareness raising campaign inviting residents to give their opinions on the role of alcohol in their lives and society. A marketing company branded and promoted the campaign between May and August 2008. Awareness was raised through advertising (for example, on buses) and engagement with the local media. Publicity and promotion included launching the campaign using the cast of a local TV soap opera, and incentives such as reduced entry to Gunther von Hagens' Body Worlds 4 exhibition. Regional television and radio stations ran the story; features were timed to be in conjunction with local road shows. These featured branded taxi cabs where people could offer opinions via 'taxicam' (closed circuit TV in the cab).

### Survey design

The survey, part of the BDD, was conducted using paper and online forms distributed opportunistically to sample the population as widely as possible. Methods of distribution of the paper questionnaire included, as an insert in free local papers, in health settings such as doctors' surgeries, and in town and city centres. Paper questionnaires incorporated a detachable participant information sheet to raise awareness of the confidential and voluntary nature of the survey and to direct participants towards sources of support for alcohol misuse, if needed. A freepost address enabled easy return. The online form ran concurrently; this was publicised in all media interviews, and given as a weblink on local media websites. The form replicated the paper version, with the information sheet reproduced on the front page. Compliance was not recorded due to the opportunistic nature of the survey, with analysis focusing on relationships between variables recorded by individual participants [15]. Ethical approval was gained from Liverpool John Moores University Research Ethics Committee, with consent assumed by self-completion of the questionnaire.

### Questionnaire variables

The one page questionnaire was designed as a short form with simple questions, in order to include a wide range of educational attainment, and to be suitable for delivery in a range of settings. It captured data on socio-geodemographic characteristics (age, sex, ethnicity, postcode, income). Where possible, standard questions were used. Age and ethnicity categories were compatible with the General Household Survey and the national census [34,35]. Income questions replicated those piloted in 2007 for the census,[36] but the original eight categories were collapsed to five for reasons of space. Categories at the lower income end of the scale were preserved since we hypothesised that those with lower income may be more sensitive to price.

Alcohol questions were based on previously validated questions measuring consumption on a specific day in the last week,[37] expanded to capture consumption for the previous week. Respondents were asked 'in general, how often do you drink' (never, monthly or less, once or twice a week, three or four days per week or ~daily). Quantity of alcohol consumed in the previous seven days was derived from number of drinks entered for each drink type recorded (175 ml standard glasses of wine, pints (568 ml) of low, medium and strong beer/lager/cider, 25 ml shots of spirit, single glasses of fortified wine, and bottles of alcopops). Respondents were asked 'where does most of the alcohol you drink come from?' and to select as many as applied from the following: pubs/bars/clubs, supermarkets, off-licences, other people (parent, friends), other (e.g. abroad, mail order).

Perceptions on impact of alcohol price were gleaned by asking: 'Do you think the following would increase or decrease people's alcohol use: 1) low prices and discounts; 2) increased alcohol prices.' The options available were 'decrease', 'no change' or 'increase'. The questions were phrased in terms of the behaviour of 'people's' rather than 'own' alcohol use in order to gain an understanding of whether respondents believed price had an effect on the population. People's views about how a complex stimulus (such as price) affects their own behaviour and that of others are subject to a number of unconscious biases. Not only do people tend to project their own opinions on others (in the 'false consensus effect'[38], there is also a tendency to modify one's own belief when one knows the opinions of other (similar) people ([39]'chameleon effect'[40]). However, while people view themselves as relatively variable in terms of behaviour, there is a tendency to view others as much more predictable in their personal traits across different situations (known as trait ascription bias [41]). Thus, '...it can be easier... to get a grip on people's political thinking by investigating what they think others think as against what they themselves think.' ([42], page 93).

The questionnaire was piloted in two major regional conurbations, and adjusted prior to the main survey.

### Data analyses

Paper responses were entered manually, with one in every ten forms checked for input accuracy. Consistency checks were conducted between the online and paper datasets. A total of 30,096 adult NW residents provided at least one opinion, representing ~0.5% of the ~5 million adult population. Half (50.5%) responded via the paper form. Individuals were excluded if they missed demographic questions (2.7% omitted gender, 2.5% age) and/or were underage (< 18 years, 2.3%). Of the remaining 28,243, 80.7% (n = 22,780) consumed alcohol in the week prior to survey and provided details of quantities consumed, and of these, 99.3% (n = 22,617) answered the two alcohol price-related questions. Drink strengths and quantities were used to calculate the total alcohol consumed in the previous week [43] and converted to average grams/day.

For Pearson's chi-square analysis individuals were coded as whether they identified that low prices and discounts would increase consumption (yes/no) and price increases would decrease consumption (yes/no). Significant effects of demographic characteristics, drinking behaviours on the perceptions of the effect of price on consumption were identified.

For multinomial logistic regression analysis, combinations of answers to the price questions that were deemed logical were included: for the low prices and discount question, this was either 'increase' the amount people drink or 'no change'; for the increasing price question, 'decrease' or 'no change' were included. These combinations of possible responses accounted for 95% of the sample (table 1). Respondents were coded as believing that alcohol is 'price inelastic' if they reported 'no change' for both questions (n = 3569), 'completely price elastic' if they reported that both price conditions affected population behaviour (in the logical direction, n = 4531) and 'lower price elastic only' if only the low price condition affected behaviour (n = 12975). Few (n = 392) were in the 'high price elastic only' category (i.e. reported that price increases would decrease people's drinking but low prices caused no change) and thus excluded.

**Table 1:** Price elasticity categorisation of last week drinkers according to their perceptions of price effects

Response to price increase		Response to low prices and discounts		
		Increase	No change	decrease
Increase	Label	-	-	-
	Total number	667	226	79
	MLR total	(excluded^1^)	(excluded^1^)	(excluded^1^)
				
No change	Label	Lower price elastic	Inelastic	
	Total number	12975	3569	100
	MLR total^2^	11788	2965	(excluded^1^)
	*MLR males*	*4005*	*1197*	
	*MLR females*	*7783*	*1768*	
				
Decrease	Label	Completely elastic	High price elastic	
	Total number	4531	392	78
	MLR total^2^	4210	(excluded^1^)	(excluded^1^)
	*MLR males*	*1863*		
	*MLR females*	*2347*		

Price questions were 'Do you think the following would increase or decrease people's alcohol use: 1) low prices and discounts; 2) increased alcohol prices.' The options available were 'decrease', 'no change' or 'increase' (n = 22617).

MLR--included in Multinomial logistic regression (n = 18963). ^1^Category excluded from multinomial logistic regression model. ^2^Those with income 'unknown' were excluded from multivariate model.

The three outcome categories were subject to a forward stepwise multinomial logistic regression, with belief in alcohol as 'price inelastic' as the reference point. Predictor variables were age, gender, ethnicity (categorised as white and not white), income, drinking level and location of purchase (restricted to the two major locations identified: supermarkets, 68.5%; pub/bar/club, 45.1%; each coded yes/no). Income and Index of Multiple Deprivation (IMD: an area-based measure derived from the postcode) showed similar distributions (IMD being the inverse of income), but income was selected as the predictor since completion was higher (1935 cases where income was missing vs 6558 postcode incomplete). After inspection of interactions, variables that combined drinking levels with income were included in the model. The model identified independent relationships between demographics and behaviours and perceptions of the effect of price on population behaviour describing adjusted odds ratios (AOR) with 95% confidence limits. Significance was assumed at the 5% level. SPSS v17 was used for analyses.

### Sample characteristics

Of the analysed sample (n = 28,243), females were overrepresented (63.9% of sample vs 51.6% of north west population). The sample overrepresented younger people (68.4 of the sample were aged between 25 and 54, while 51.2% of the population falls in this range) with those aged 75+ years (9.7% of the population) contributing the fewest responses (2.5%). The sample was representative in terms of the proportion of respondents who were white British (92.8%, compared to the official population estimates for north west England [44]: 92.2%), white Irish (1.45% cf 1.15%), white European (1.35% cf 1.11%), black/black British (0.67% cf 0.62%), Chinese (0.29% cf 0.40%) and mixed race (0.98% cf 0.93%). However, Asian/Asian British were under-represented (1.37% of the sample compared to official estimates of 3.42%). Of the five counties in north west England, the rural county of Cumbria and the urban metropolitan area of Greater Manchester were ~20% over represented in the sample, the county of Lancashire (a mix of industrial towns and rural) was 33% underrepresented and Merseyside and Cheshire counties were represented in proportion to their population size.

## Results

### General characteristics

Among respondents providing details of their consumption frequency (n = 27,194), 9.0% reported that they had never drank alcohol, 21.1% did so monthly or less, 39.4% once/twice a week, 19.5% three or four times each week, and 11.1% reported that they drank almost daily. Gender was associated with drink frequency (χ2 test for trend = 611.8, df = 4, p < 0.001), with 16.6% of males drinking almost daily compared with 7.9% of females. Few (~6%) in the youngest age category never drank or--at the other extreme--drank almost daily. Drink frequency was more dispersed among the elderly (75+ years), and this age category contributed the highest proportion declaring that they never drank (25.9%) and also that they drank almost daily (18.2%). The mean quantity of alcohol consumed for males and females was 17.6 g (95% confidence intervals, CI; 17.24-18.08 g) and 10.4 g (95%CI; 10.21-10.53 g) per day respectively. (The mean and CI were calculated from the natural logarithm - to correct for skewed distributions - and then transformed back to the original scale to present here.) Over one fifth (21.2%) of respondents consumed 1-10 g a day (inclusive), and 18.2% consumed 21-30 g. A minority (2.6%) claimed they consumed ≥ 71 g a day. The percentage of the population drinking at levels that could harm their health varied from 4.8% (95%CI 3.8,5.9) in the predominantly rural county of Cumbria to 7.5% (95%CI 6.9, 8.1) in the urban county of Greater Manchester (age- and sex-standardised estimates of the percentage drinking more 400 g alcohol per week for males or 280 g for females).

### Responses to questions on price

Among last week drinkers who answered the price questions (n = 22,617), 80.3% believed low prices and discounts would increase the amount of alcohol people consumed (table 2). Females were more likely to hold this view than males, whilst older age categories (65+ years) were less likely than other age groups to believe low prices would increase consumption. There was no significant difference between ethnic groups. Compared with respondents on the lowest annual personal income (< £4,000), those earning £37,000 or more were more likely to indicate that low prices caused people to drink more. The reported quantity of alcohol consumed in the last week by respondents had the strongest association with low price impact: 83.5% of respondents who drank 1-5 g/day agreed that low prices increased consumption, compared with 71% of respondents who drank > 80 g/day. Those mainly purchasing alcohol from supermarkets were more likely to agree that low prices and discounts increased people's drinking, while this was reversed for those buying predominantly from pubs.

**Table 2:** Percentage of last week drinkers who are price elastic by demographics, drinking level and alcohol source

Variable	n	Low prices/discounts	**χ**^**2**^	P	Increasing prices	**χ**^**2**^	P
**Gender**							
Male	8452	78.0	46.1	< 0.001	26.3	135.2	< 0.001
Female	14165	81.7			19.6		
**Age**							
18-24	2609	80.6	19.4	0.004	29.7	112.7	< 0.001
25-34	5019	80.4			22.7		
35-44	5488	80.3			20.8		
45-54	5074	81.1			20.9		
55-64	2963	80.8			20.6		
65-74	1022	76.2			18.4		
75+	442	75.8			19.7		
**Ethnicity**							
White British	21242	80.6	13.3	0.065	21.9	17.3	0.015
White Irish	330	77.9			23.0		
White European	293	75.1			25.6		
Black/black British	132	73.5			24.2		
Asian/Asian British	141	76.6			28.4		
Chinese/Chinese British	46	82.6			41.3		
Mixed Race	211	78.7			21.8		
Other	222	78.4			25.2		
**Income**							
Under £4,000	1014	76.8	49.0	< 0.001	29.8	55.6	< 0.001
£4,000 to £7,999	1196	77.3			21.5		
£8,000 to £16,999	4161	80.2			22.5		
£17,000 to £36,999	9592	81.0			21.1		
£37,000 or above	4719	82.3			23.5		
unknown	1935	76.3			19.2		
**Alcohol per day**							
1-5 g	4802	83.5	139.0	< 0.001	21.8	3.8	0.710
6-10 g	4107	82.7			21.7		
11-20 g	5859	81.0			22.7		
21-40 g	5025	78.2			22.3		
41-60 g	1636	74.7			21.5		
61-80 g	593	75.2			23.8		
> 80 g	595	70.8			20.8		
**Main source of alcohol**^**1**^							
Pubs/clubs No	12410	81.5	23.6	< 0.001	20.6	36.7	< 0.001
Pubs/clubs Yes	10207	78.8			24.0		
Supermarket No	7129	78.8	15.8	< 0.001	23.7	14.6	< 0.001
Supermarket Yes	15488	81.1			21.4		
Off-Licence No	18469	80.9	16.5	< 0.001	21.9	2.1	0.150
Off-Licence Yes	4148	78.1			23.0		
Restaurant No	18098	79.9	12.7	< 0.001	22.5	7.3	0.007
Restaurant Yes	4519	82.2			20.6		
Other No	21543	80.2	6.0	0.015	22.1	0.7	0.680
Other Yes	1074	83.2			21.6		
**Total**	22617	80.4			22.1		

Price elastic is defined as believing that low prices and discounts increase people's drinking and/or that high prices decrease people's drinking.

^1^Respondents could select more than one drinking source.

Overall 22.1% of last week drinkers who answered the price questions ticked 'decrease' when asked about the effect of raising the price of alcohol (table 2). Females were more likely to report that high prices would reduce consumption. The largest differential in the percentage selecting 'decrease' was across age: those in the youngest age category (18 to 24 years) were significantly more likely to agree (29.7%) compared with the oldest (75+) category (19.7%). Persons of Chinese ethnicity were more likely to agree with this statement compared with white British participants. Chinese ethnicity had a strong effect on the overall significance (the chi square value excluding persons of Chinese ethnicity was non-significant, at 7.47, df = 6, P = 0.280), but since there were very few Chinese participants (46, 0.2% of sample) this limited the interpretation of this finding. Those on the lowest annual income (< £4,000) were the most likely to agree (29.8%). Those in the other income categories were less likely to agree (all less than 24%) but response to this question was not linear. Those predominantly buying alcohol from pubs, bars and/or clubs were more likely to think a price rise would decrease consumption. There was no significant relationship between current levels of alcohol consumption and perception that a price rise would reduce consumption.

**Figure 1:**
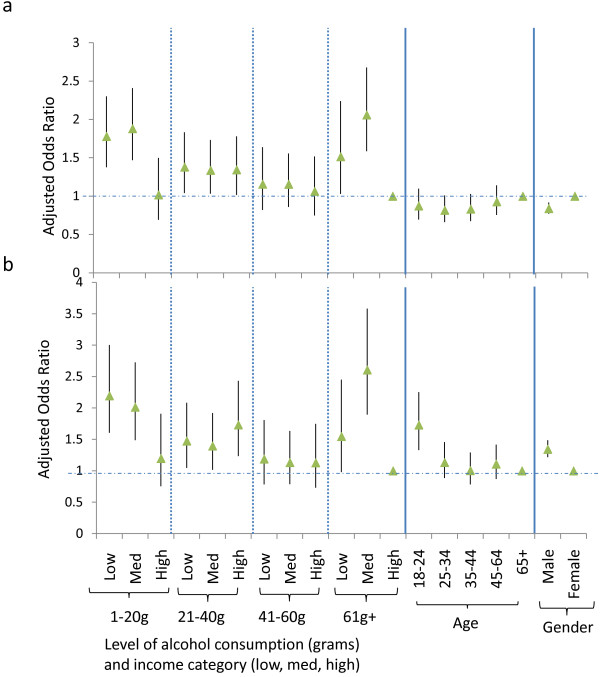
**Adjusted odds of price elasticity by gender, drinking category and income**. Results of a multinomial logistic regression model (n = 18963), showing the adjusted odds of lower price elasticity (a) and complete price elasticity (b) compared to a reference category of price inelasticity, subdivided by significant variables (age, gender and combined drinking and income categories)

Using multinomial logistic regression, gender was found to be significant predictor of price elasticity category. The interaction between income and drinking level was also significant, while variables rejected during the forward stepwise procedure were income and drinking level as separate variables, ethnicity and location of purchase. Age was retained in the model as it was a significant predictor of being completely price elastic, although not of being lower price elastic. Those in the highest income bracket who drank the most alcohol (> 60 g/day) were the least likely to be completely price elastic or lower price elastic only (compared to inelastic: definitions in table 1), and were therefore selected as the reference category. Those on low and medium income from the lightest drinking category (low income: adjusted odds ratio AOR = 1.78, 95% confidence intervals CI 1.38-2.30, p < 0.001; medium AOR = 1.88, CI 1.47-2.41, P < 0.001) and heaviest drinking category (low income AOR = 1.52 CI 1.03-2.24, P = 0.036, medium AOR = 2.06, CI 1.59-2.68, P < 0.001) were most likely to be lower price elastic (Figure 1a). In addition, all income categories of the 21-40 g/day drinking group were more likely to be lower price elastic (p < 0.05). Compared to a baseline reference category of high income/high drinking level, those with low income (AOR = 2.20, CI 1.60-3.00, P < 0.001) and medium income (AOR = 2.01 CI 1.49-2.73, P < 0.001) from the lightest drinking category were more likely to be completely price elastic (Figure 1b). Compared to the heaviest drinkers with high income, the heaviest drinkers with a medium income were significantly more likely to be completely elastic (AOR = 2.61, CI 1.90-3.58, P < 0.001) drinking categories. In addition, all income categories of the 21-40 g/day drinking group were more likely to be completely elastic (p < 0.05).

Females were more likely than males to be lower price elastic only (65% vs 57%) whilst this was reversed for complete elasticity (females 20%, males 26%, χ^2 ^= 154.7, df = 2, P < 0.001). Gender remained significant in the multivariate models (Figure 1), with males having significantly lower odds of being lower price elastic (AOR = 0.84, CI 0.77-0.92, p < 0.001), and higher odds of being completely elastic (AOR = 1.34, CI 1.22-1.49, p < 0.001). Compared to the oldest age group (> 64 years), the youngest (aged 18-24 years) were significantly more likely to be completely elastic (AOR = 1.73, CI 1.33-2.25, p < 0.001). However, age was not a significant predictor of being lower price elastic.

## Discussion

This paper provides valuable information on the opinions of the public on the impact of price on alcohol consumption. The population sampled considered that low prices and discounts encouraged greater drinking (80%), while few believed price increases would reduce drinking (22%). Research in marketing theory confirms that price is a complex stimulus. At its simplest, increased price is expected to reduce the probability of a product being purchased, but it can also have the opposite effect if price is perceived to link to quality. Another tendency is 'sale proneness', defined as an increased propensity to respond to a purchase offer because of the sale form in which the price is presented. Thus, an item becomes more attractive for purchase when the price is presented in the form of a sales offer, compared with presentation at the standard price [45].

We addressed what respondents thought would be the effect of low prices and discounts and increased alcohol prices on 'people's alcohol use', rather than their perceived impact on their own alcohol intake. While this supports the intent to measure a population-level effect, responses varied by demographic, socioeconomic and drinking habit, making it seem likely that respondents projected views on other similar populations in a 'false consensus effect' [38]. We thus conjecture that the beliefs expressed relate to participants' own anticipated reactions to the two potential pricing strategies. We acknowledge that beliefs about these behaviours do not necessarily translate into actual behaviour.

It might be expected that there would be a balance of agreement between both price statements, and insensitivity to increasing price would be incongruous. Some groups exhibited this disparity more than others: females were more likely to view alcohol as 'lower price elastic only', believing that low prices and discounts increased people's drinking but that price increases had no effect. In contrast, males were more likely than females to be completely price elastic. Modelled UK data have suggested that because women purchase more alcohol from the off-licensed trade (where strategies such as minimum pricing are more likely to have an impact) and because women consume more expensive drinks, they are more likely to be affected by increased prices than men [46]. We hypothesise that women in our survey may have been more likely to believe that household budgets would be compromised in order to maintain consumption.

The youngest age group were more likely to be completely price elastic, suggesting that age may have an effect on the degree to which the consumption of alcohol is an entrenched behaviour. Trends over the last decade in alcohol consumption in this age group show fluctuating levels compared to more stable patterns in older ages,[47] suggesting that behaviour, as well as perceptions, may be more flexible in younger people.

Sensitivity to price reductions has been strongly supported in the literature, particularly where tax reductions have facilitated cheaper alcohol, such as in Finland and Switzerland [11,48,49]. In the UK, consumers are regularly presented with alcohol at discounted prices, including those that encourage bulk purchase (for example, volume discounting) [14]. Thus, individuals make choices influenced by low prices, which may lead to greater awareness of their impact.

Lack of perception that price increases would reduce drinking is an interesting observation that may relate to the UK drinking population having little experience of rises in alcohol prices--the price of alcohol in real terms having fallen consistently over the past three decades [13]. In fact the small incremental rises associated with annual government budget changes have been largely met by alcohol retailers who sell alcohol at below cost price, protecting the public against experience of higher costs [14,50]. However, prices in on licence setting have increased and people who listed pubs, bars and clubs as a main alcohol source were more likely to think price increases would decrease intake. Sensitivity to price increases are reported in the international literature, indicating the likelihood that price rises do result in reduced drinking;[7-9] a minimum price for a unit of alcohol (10 ml or 8 g of alcohol), now under discussion in a number of countries, are thus still anticipated to be a major measure to curb alcohol consumption.

An alternative interpretation, not widely evidenced in the literature, is that people consider relatively high levels of alcohol to be an essential component of daily life, such that they view people as willing to absorb the increased cost of alcohol rather than reduce their drinking. One study in Sweden noted that where price increases were only attached to specific alcohol types, consumption of cheaper products rose [51]. In our survey, the heavier drinkers were least likely to report that low prices increased consumption, suggesting true price insensitivity. However, those in the highest drinking category appeared strongly influenced by income, with the low and medium income heavy drinkers being more likely to be price elastic. In contrast, income levels in the more moderate (21-40 g/day) drinking category did not influence elasticity. Further, those on low incomes and who drink least were more likely to believe increased prices will decrease consumption. This is consistent with alcohol playing a less important role in their lives and the relative impact of increased prices being greater. Other studies counter this, suggesting harmful drinkers are more sensitive to price [48,52,53]. Modelling work has suggested a minimum price of £0.50 (€0.56; $0.83) would lead to a 10.3% decrease in consumption by harmful drinkers compared with 6.9% across the population [3].

Our findings were generated from a large survey (> 29,000 people), ~0.5% of the adult population in NW England. As a convenience sample, not selected randomly, it may not be fully representative and the findings from the inferential statistics should be interpreted with caution. Large-scale convenience samples are useful when the random sampling is not possible (e.g. because of a lack of a sampling frame [54]) or when there is an existing large convenience sample making the additional cost of random sampling hard to justify [55,56]. Random sampling was not appropriate for the Big Drink Debate, which sought to give all residents the opportunity to participate and did this via widespread advertising and the use of a variety of methods to engage participants in different settings. This, coupled with the simplicity of the survey tool, provided wide recruitment and the final sample included a breadth of public involvement. This was highlighted by the broad distribution of wealth and age in the categorised populations, and the full representation of poorer and less educated populations, who are often missed in such surveys. However, males, in particular, were underrepresented. Those of Asian and Asian British backgrounds were also less likely to participate. Since 70% of Asian people in the UK belong to religious groups that discourage alcohol use (i.e. are either Muslim or Sikh [44]), the Asian people exposed to the survey may have felt it was less relevant to them. Indeed, the small proportion of non-drinkers suggest drinkers were more inclined to participate (elsewhere, NW estimates suggest that 22% of the population are non-drinkers compared with 9% here);[57] although because this study focuses solely on the opinions of those who drink, the findings are not compromised. Capture of information in the many different settings required a short simple questionnaire which reduced our ability to generate in-depth intelligence on both alcohol consumption and surrounding beliefs. Surveys are subject to respondents' recall (where alcohol consumption can interfere with memories held)[58] as well as honesty, and have been shown to produce considerable under-estimates of total amounts consumed [59]. Although respondents were assured of their anonymity, we assume the reported quantities consumed are a low estimate.

## Conclusions

Much of the damage relating to the lack of control over alcohol pricing has already been done. The vast majority of persons surveyed believe that price reductions increase alcohol consumption; consistent with the real term reductions in price seen over recent decades and corresponding to the observed rise in consumption [12,13]. The continued focus of the alcohol industry in using special offers including 'buy one get one free' encourages greater consumption, as known from marketing theory [45] and confirmed by our respondents. International evidence shows that price is an important way to impact on the drinking behaviour of the population,[4,12,52] with a minimum pricing policy thought to be most effective in order to ensure all retailers pass increased prices to the consumer [3,5,17]. Despite the fact that the population has not had experience of increasing alcohol prices on which to base opinions on the effect of increasing price on drinking behaviour, a significant minority of survey respondents do perceive higher prices will reduce drinking. However, the instigation of such a policy relies on strong action from government, and is unlikely to happen without more support from the public. More work is required to enable the public to 'buy into' the belief that price increases would impact on the population's drinking behaviour and ultimately on alcohol related harms. However, those planning public health interventions also need to recognise that alcohol is an addictive drug, and that the UK population as a whole has become habituated to a high level of consumption: even if prices are increased, people may, to a certain extent, sacrifice other parts of their expenditure to maintain consumption at levels they now feel they need. Our survey, which sought the views of over 20 000 people, supports this by suggesting the population may be reticent to reduce their drinking when prices increase.

## Competing interests

The authors declare that they have no competing interests.

## Authors' contributions

PAC contributed to the questionnaire design, in collaboration with the Government Office North West, and led the analysis and interpretation and produced the first draft of the paper. PAP-H and MAB contributed to analysis, interpretation, refining the paper and synthesis of policy implications. MM helped draft the paper. CH assisted with statistical analysis and drafting the paper. LB carried out multinomial logistic regression analysis. All authors read and approved the final manuscript.

## Pre-publication history

The pre-publication history for this paper can be accessed here:

http://www.biomedcentral.com/1471-2458/11/664/prepub
